# Diode laser posterior nasal neurectomy with or without partial posterior turbinectomy in patients with intractable vasomotor rhinitis: a prospective comparative study

**DOI:** 10.1007/s10103-026-04805-8

**Published:** 2026-02-10

**Authors:** Sherif Askar, Ashraf Elmalt, Ahmed Sherif

**Affiliations:** Zagazig University, Zagazig, Egypt

**Keywords:** Diode laser, Posterior nasal neurectomy, Intractable rhinorrhea

## Abstract

**Purpose:**

To assess and compare the surgical outcomes of the diode laser posterior nasal neurectomy with or without posterior turbinectomy in patients with refractory vasomotor rhinitis, regarding rhinorrhea, obstruction and irritation symptoms.

**Methods:**

The study group (32 patients) was distributed into two equal groups: Group A, diode laser posterior nasal neurectomy with posterior turbinectomy group (LPNT), and Group B, diode laser posterior nasal neurectomy only (LPN). In DLT, the machine was in continuous mode with intermittent loading, with the laser energy level set to 6 W.

**Results:**

In the 6th month, in group A, there was a highly significant difference (in comparison to preoperative data) regarding rhinorrhea and nasal obstruction (*P* < 0.0001). In group B, a significant difference between the pre- and postoperative findings regarding rhinorrhea and nasal obstruction **(***P* < 0.05) were reported. Regarding rhinorrhea and nasal obstruction, a highly significant difference between the two groups was reported. On comparison of postoperative data of both groups, a significant difference was reported regarding nasal irritation symptoms (*P* < 0.05**)**. Methylene blue saccharine test was applied to test the mucociliary clearance system objectively; the mean of both groups (in the 6th month) showed a statistically significant difference with a tendency towards group B. The Visual Analog Score showed significant improvements in both groups.

**Conclusion:**

Laser ablation of the posterior nasal nerve has proved to be a reliable, safe, and efficient technique. The combination of laser posterior nasal neurectomy and conservative posterior turbinectomy allowed the reduction of nasal hyperreactivity and improved the annoying symptoms of refractory rhinorrhea.

## Introduction

Vasomotor rhinitis (VMR) is the most common type of non-allergic rhinitis (NR). VMR is a clinical diagnosis and is considered when the patient has chronic symptoms of nasal obstruction and/or rhinorrhea, not related to a known specific antigen/infectious agent, without a definite etiology after a thorough search for a diagnosis. The specific pathophysiology of VMR is yet to be discovered; parasympathetic/sympathetic imbalance was suggested. The best treatment option is the avoidance of the trigger agent; medical therapy is an adjunct. Surgical intervention is a valuable option when others fail [1–3].

A wide variety of surgical maneuvers are currently available, including turbinate reduction and neural jeopardizing maneuvers. Turbinate reduction can alleviate obstruction symptoms. Neural ablation began with vidian neurectomy, which aimed to disturb the autonomic nerve supply to the nasal mucosa. With the advent of naso-endoscopy, the more conservative posterior neurectomy (PN) was described to ablate the posterior nasal nerve, which is a peripheral branch of the sphenopalatine ganglion and is composed of postganglionic pterygoid nerve/maxillary nerve sensory fibers. The nerve contains multiple groups of nerve fascicles that pass to the nasal cavity through the sphenopalatine foramen. PN is a highly selective neurectomy that can effectively manage intractable rhinitis and minimize the complications of vidian neurectomy.

Although different PN techniques are described in the literature, selecting the suitable one for the individual patient is based on the surgeon’s/patient’s preference, budget, and hospital facilities. The ideal procedure for PN should preserve the physiological functions of the nose and should be associated with no complications. Unfortunately, such a procedure remains elusive, and no one is perfect [4–8].

We hypothesize that the diode laser (DL) suits PN and turbinate reduction in patients with refractory vasomotor rhinitis. It could relieve patients’ symptoms while sparing the mucosa’s physiological role. Moreover, we believe that Laser surgery has many advantages; limited tissue trauma and less bleeding would minimize complications [9]. Also, we assume that combining laser PN and partial posterior turbinectomy would result in better control of rhinorrhea (and other symptoms of rhinitis) than PN alone.

The current research aimed to assess and compare the surgical outcomes of the diode laser posterior nasal neurectomy with or without posterior turbinectomy in a selected group of patients with chronic refractory vasomotor rhinitis, regarding rhinorrhea, nasal obstruction and irritation symptoms.

## Patients and methods

### Study design

This prospective randomized controlled interventional study was conducted on adult patients with chronic rhinitis for more than 6 months (who did not respond to medical treatment for at least 4–6 months). The study group had the following nasal symptoms (rhinorrhea, nasal obstruction, and sneezing); patients with persistent extra-nasal symptoms (e.g., ear, facial fullness, psychological upsets, chest problems, and chronic cough) were not included. Patients were chosen from the outpatient clinic and were managed between June 2020 and May 2025. Assuming the probability of symptom persistence in the diode laser posterior nasal neurectomy with posterior turbinectomy group (A) is 46.7%, in the diode laser posterior nasal neurectomy only group (B) 39%, confidence level 95% and power 80% (estimated β = 20), so, the total sample size was 32 patients (16 in each group). After the explanation, the subjects gave written informed consent, which was approved (IRB: 1666) (Table 1).

**Table 1:** Rhinorrhea pre- and post-operative assessment

Rhinorhea	Group A		Group B		*P* value
preoperative	No	%	No	%	
Yes	16	100.0%	16	100.0%	……
one month					
Yes	16	100.0%	16	100.0%	….
three month					
No	10	62.5%	10	62.5%	1.000
Yes	6	37.5%	6	37.5%	
six months					
No	15	93.8%	15	93.8%	1.000
Yes	1	6.3%	1	6.3%	
Overall Postoperative improvement					
Cochran’s Q	40.96		40.96		
P value	0.000		0.000		
	P value		P value		
preoperative versus 3 months postoperative	0.002		0.002		
preoperative versus 6 months postoperative	0.000		0.0000		

### Exclusion criteria

Patients with a history of nasal surgery, severely deviated nasal septum, and nasal polyps/tumors were excluded from the study. Other exclusion criteria included diabetes mellitus, asthma, coagulation disorders, uncontrolled hypertension, and cardiac pacemakers. Smokers were excluded from the study.

### Methods

A detailed history and a general examination were the starting point of this study. The Visual Analog Score (VAS-s) was answered by the study group; the score assesses the severity of the individual patient’s symptoms (rhinorrhea, nasal obstruction, and sneezing) from 0 to 5 as: mild (scores of 5–10), moderate (scores of more than 10 to 15), or severe (scores of more than 15) [10].

Complete ENT examination followed. Nasal endoscopy (0º endoscope, Karl Storz-Endoskope, Tuttlingen, Germany) was done after local decongestion for a few minutes (Epinephrine 1 mg/ml ampoule, Chemipharm Pharmaceutical Industries-SAE, 6th October, Giza, Egypt). 1 mg of ephedrine in 200 ml saline solution (Allmed Middle East, 6th October-Giza, Egypt) makes 1/200 solution. Methylene blue saccharine test (MBST) was applied to test the mucociliary clearance system objectively (Methylene blue (MB): Egyptian Diagnostic Media-Cairo; Egypt-Saccharine (Sc): sodium saccharine 98% by China Pingmei Shenma Group, Kaifeng Xinghua Fine Chemical Co, Ltd., Kaifenghsien, Henan, China). The test was done by putting the saccharine (mixed with MB) at the anterior end of the inferior turbinate. When the patient could detect the sugar taste and the blue color (of the dye) could be visualized at the oropharynx, the test time was documented [9].

Included subjects were randomly distributed (the closed envelopes employed randomization with letter A for Group A and letter B for Group B) in two groups: group (A) would have diode laser posterior nasal neurectomy with partial posterior turbinectomy (LPNT) group and group (B) were scheduled for diode laser posterior nasal neurectomy only (LPN). A third control group (of volunteers) was included for the MBST only. CT scans of the nose and paranasal sinuses were done for all patients (not for the controls). Routine preoperative investigations would follow. In the theatre, laser precautions were strictly followed to avoid harm to all attendees (Table 2).

**Table 2:** Saccharine test results pre- and post-operative assessment

saccharine test (minutes)	Control group		Group A		Group B		*p* value
	Mean	SD	Mean	SD	Mean	SD	
preoperative	7.93	1.59	15.56	1.590	16.31	1.740	P1, P2 < 0.001 P3 = 0.213
1 month postoperative (minutes)	7.93	1.59	18.00	1.673	18.19	2.040	P1, P2 < 0.001 P3 = 0.778
3 months postoperative (minutes)	7.93	1.59	14.19	1.682	12.84	1.64	P1, P2 < 0.001 P3 = 0.027
6 months postoperative (minutes)	7.93	1.59	12.63	1.360	11.00	1.633	P1, P2 < 0.001 P3 = 0.005
the difference within the same group			P value		P value		
preoperative versus 1 month postoperative			0.000		0.000		
preoperative versus 3 months postoperative			0.000		0.000		
preoperative versus 6 months postoperative			0.000		0.000		

P1: the difference between Control group and Group A; P2: the difference between Control group and Group B, P3: the difference between group A and Group B

### Operative technique

Surgical interventions were performed under general endotracheal anesthesia with a cuffed endotracheal tube and wet pharyngeal pack (Ultra for Medical Products Co, Abnoub, Assiut-Egypt). The patient was placed in the supine position (a reverse Trendelenburg position) with the head elevated to 30⁰ to minimize bleeding by decreasing venous return. The endoscopic nasal examination was done first. A local infiltration (epinephrine in saline 1/200 solution) was injected inferior to the posterior attachment of the middle turbinate (just behind the posterior fontanelle using a 22-gauge spinal needle, with the needle tip bent to 45⁰.

**Fig. 1:**
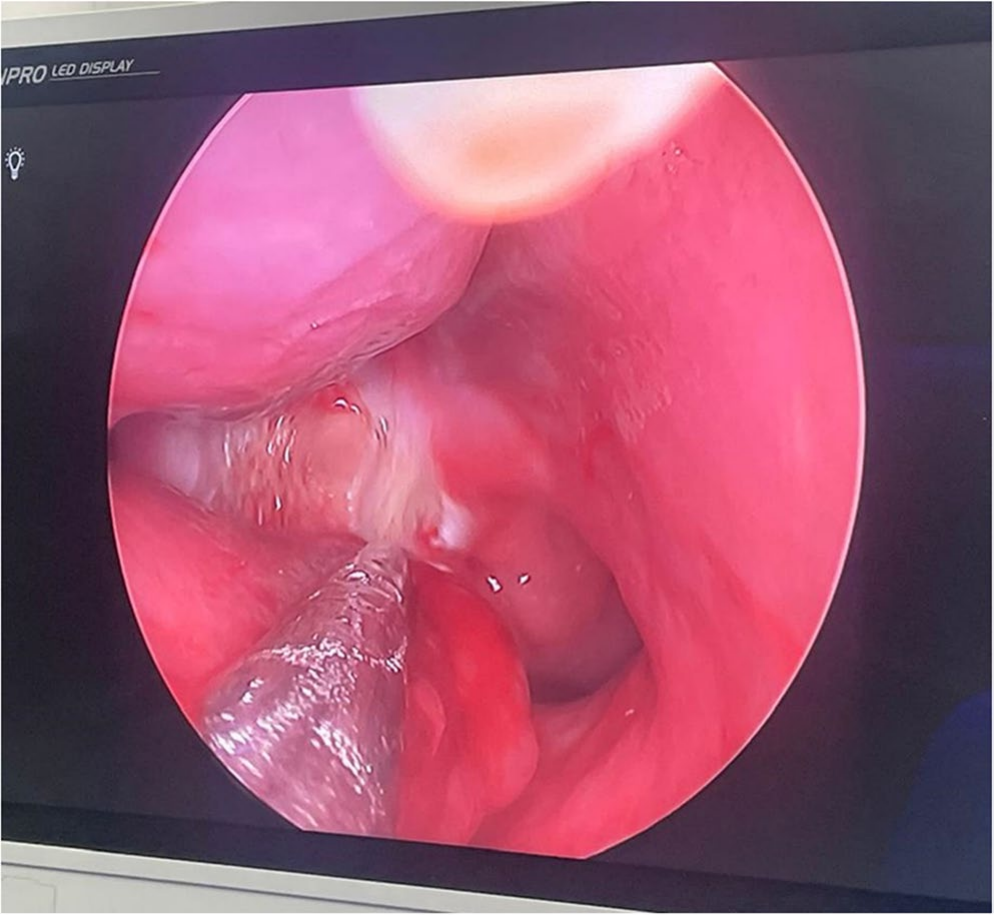
Intraoperative view of laser posterior neurectomy; *Note*: submucosal posterior turbinectomy

In group A (LPNT group) the diode laser probe was introduced into the nose under endoscopic visualization connected to a video camera (Karl Storz; Endoskope telecam- DX pal-202320 20; Tuttlingen-Germany). The diode laser machine (V-100 serial No SN: GA13V872, Wuhan Gigaa Optronics Technology Co, Ltd, Wuhan-China) has a wavelength of 810–1000 nm. The laser mode was continuous operation with intermittent loading. The laser energy level was set to 6 W. The laser fiber was Bare Laser Fiber (GA-400-1-Gigga Optronics Technology Co Ltd, Wuhan-China. The fiber probe was placed in the target area, 0.5 × 0.5 cm^**2**^ immediately below the posterior end of the middle turbinate (at the anatomical location of the posterior nasal nerve), and then, the laser was applied (ablating mucosa and submucosa directly to the bone) with slow retraction of the probe for 10 s/cm, so a total energy of 60 J/cm was delivered. The area above the choana was also ablated to deactivate the septal branch (Fig. 1).

Then, the probe was inserted submucosally in the posterior third of the inferior turbinate (about 1.5 cm) and set to ablate the tail of the inferior turbinate. The same technique was used on the other nasal side. The total laser energy was about 160 J/side. In group B, LPN only was performed.

By the end of the surgery, the nasal cavity was packed with thin cotton packs (epinephrine in saline 1/200 solution), which was removed 2–3 h postoperatively. Postoperative pain was effectively controlled with paracetamol, 500 mg tabs/8 h. Nasal saline irrigation was ordered 3–4 times daily. The operative time was the time taken to complete the planned procedure only. The time for induction and recovery of anesthesia was not counted.

### Post-operative care

Patients were discharged at night and followed up every 7 days during the 1 st month (with cleaning of dry crusts) and then monthly for one year. During follow-up visits, symptom evaluation (nasal obstruction, rhinorrhea and sneezing) was reported with VAS-s. During the 1 st, 3^rd,^ and 6th months of visits, nasal endoscopy and MBST were performed. Results were collected and tabulated.

### Statistical analysis

Patients’ data were collected, tabulated, and statistically formulated using SPSS 22.0 for Windows (SPSS Inc., Chicago, Illinois, and Microsoft Office Excel 2020 for Windows (Microsoft Cor, Redmond, WA-USA). Continuous data were expressed as mean ± SD & median (range). The categorical data was expressed as numbers (percentages). Continuous variables were checked (for normality) by the Shapiro-Wilk test. The Wilcoxon signed ranks test was applied to compare two dependent groups of non-normally distributed data. The Chi-square (χ2) test was applied to compare the percent of categorical variables. All tests were two-tailed. The power analysis estimation for the sample size was 80%, the confidence interval (CI) was 95%, and the margin of error was 5%; *P* < 0.05 was significant, P ˃0.05 was non-significant, and *p* < 0.001 was considered highly significant. Kruskal Wallis was used (as a non-parametric test) to compare more than two groups.

## Results

The study group included thirty-two patients (18 females and 14 males) with an age range of 20–36 years (mean = 25.0 ± 3.06). Patients were randomly distributed in two equal groups (16 in each). Group A (LPNT) included 10 females and 6 males with a mean age of 22 ± 3.22, while group B (LPN) included 8 females and 8 males with a mean age of 23 ± 4.51. Comparing the means of age in both groups, the difference was not statistically significant (*P* = 0.1584; t = 2.0516; 95% confidence interval: −6.4620 to 1.7361). There was no statistically significant difference between the groups regarding the preoperative findings (*P* > 0.05). A third control group (9 females and 5 males; mean age = 23.5 ± 4.1) was assigned for MBST only. The follow-up period ranged from 13 to 19 months (mean = 15 ± 2.08).

The subjects included had refractory bilateral rhinorrhea for more than 6 months. Nasal obstruction and irritation symptoms (sneezing) were mentioned by 29 patients (group A: 15; group B: 14). None of them had severe nasal septum deviation or nasal polyps. No patient had a history suggestive of allergy. Documented results of the skin-prick test and radioallergosorbent test (for a specific allergen) were negative for all patients; hence, the study group had a diagnosis of vasomotor rhinitis. The mean operative time for LPNT (group A) was 19–25 (mean = 22 ± 1.02) minutes, while it was 14–20 (mean = 16 ± 2.31) minutes for the LPN (group B) (*p* = 0.0004, t = 4.2381 and 95% confidence interval of this difference were from 0.8971 to 3.1105). In all patients, no significant bleeding was reported during the surgery.

During the 1 st month, there was no significant difference between the studied groups’ postoperative complications regarding pain, crusting, and synechia (*P* = 0.135). No major complications (orbital injury, severe epistaxis) were reported. By the 3rd month, there were no reports of crusting or nasal synechiae (in either nasal cavity).

**Fig. 2:**
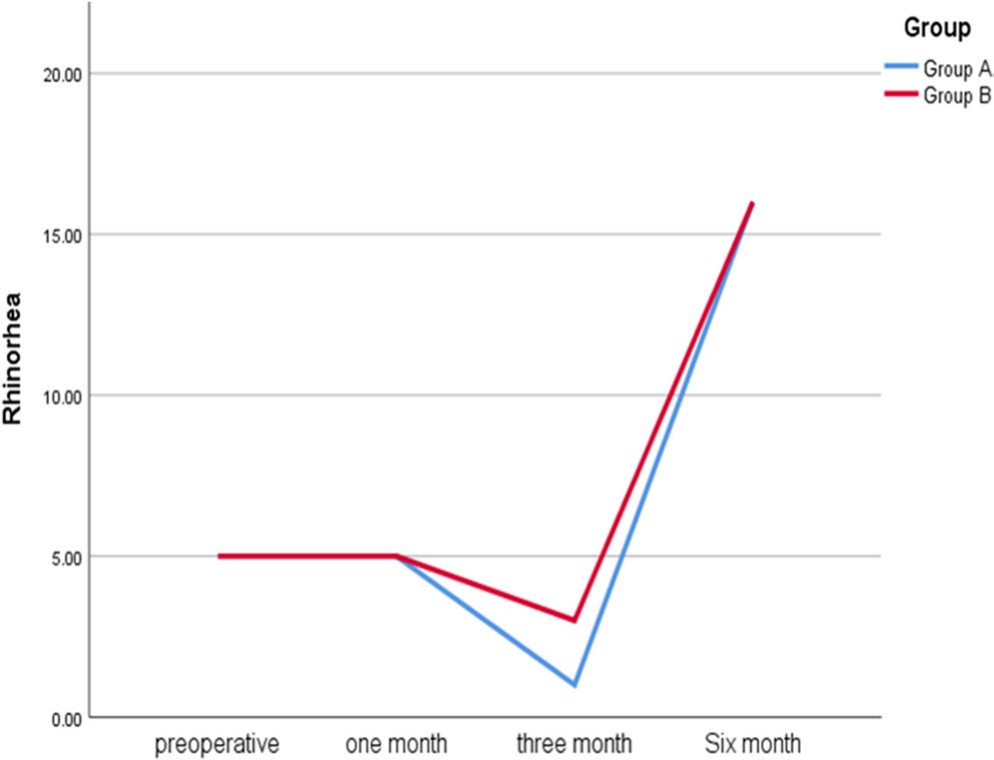
The comparison between both groups regarding rhinorrhea

Regarding rhinorrhea, it was consistently present in both groups preoperatively and at one month postoperatively (100%). However, significant differences emerged at three months, where 81.3% of Group A had no rhinorrhea compared to 18.8% in Group B (*p* = 1.000). By six months, 93.8% of both groups (A & B) reported no symptoms; no statistically significant difference (**χ**^**2**^ 9.264; *p* = 1.000) was reported. The Cochran’s Q test revealed significant postoperative improvement, with p-values of 0.004 for Group A and 0.008 for Group B (Fig. 2).

**Fig. 3:**
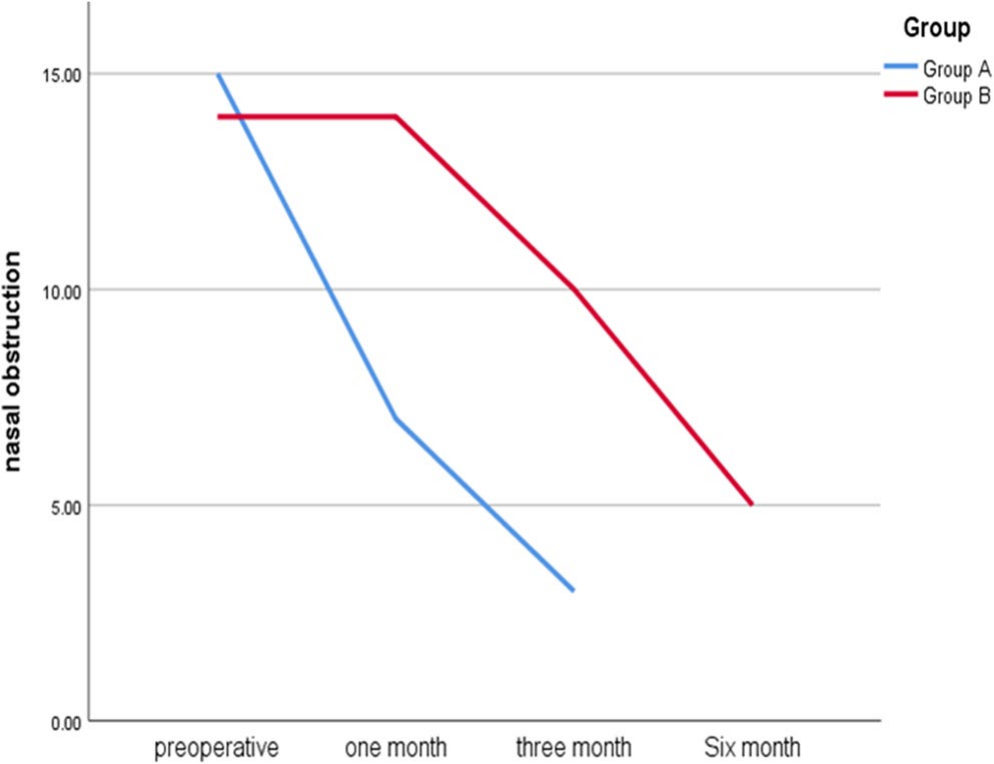
The comparison between both groups regarding nasal obstruction

The assessment of nasal obstruction revealed significant differences between Group A and Group B across various time points. Preoperatively, 93.8% of patients in Group A and 87.5% of patients in Group B reported nasal obstruction, with no statistically significant difference (*p* = 0.544). However, at one month postoperatively, a significant difference was observed, with 56.3% of Group A reporting no obstruction compared to only 12.5% in Group B (*p* = 0.023). This trend continued at three months, where 81.3% of Group A had no obstruction versus 37.5% of Group B (*p* = 0.012). By six months, all patients in Group A reported no nasal obstruction, while only 31% in Group B did (*p* = 0.043). The Cochran’s Q test demonstrated significant postoperative improvement in both groups, with p-values of 0.000 (Fig. 3).

The assessment of lacrimation symptoms demonstrated significant differences between the two groups, particularly at one month postoperatively. Preoperatively, 93.8% of Group A and 87.5% of Group B reported symptoms, with no significant difference (*p* = 0.544). At three months, no significant differences were observed (*p* = 0.719), and by six months, both groups had similar rates of lacrimation symptoms, with no significant difference (*p* = 1.000). The Cochran’s Q test revealed significant postoperative improvement (p-values of 0.000 for Group A and 0.012 for Group B).

**Fig. 4:**
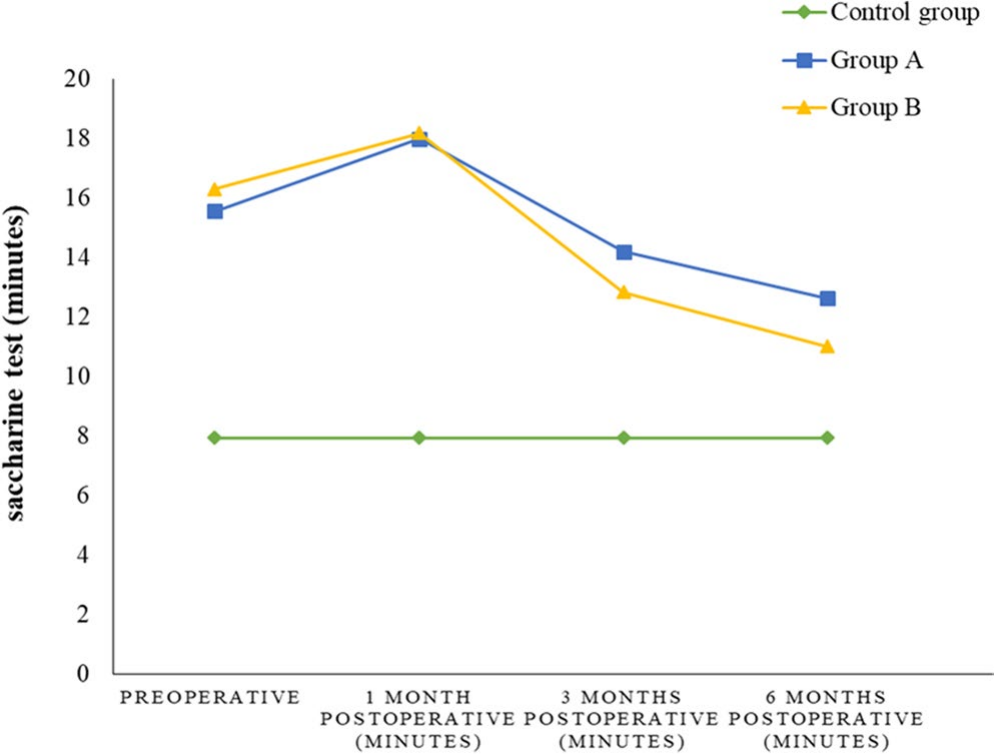
The comparison between three groups regarding methylene-blue saccharine test

Preoperatively, MBST showed no significant difference between the two groups (*p* = 0.213). At one month postoperatively, both groups experienced an increase in saccharine test times, again showing no significant difference (*p* = 0.778). However, significant improvements were noted at three and six months postoperatively. At three months, the test showed a statistically significant difference (*p* = 0.027). By six months, MBST was also statistically significant (*p* = 0.005). The differences within each group from preoperative to postoperative assessments were all statistically highly significant (*p* < 0.000). Comparing the mean of the test in the 3rd month, the difference was extremely statistically significant (p˂0.0001, t = 7.1322, 95% confidence interval was from − 2.8794 to −1.7450). The comparison of the mean of MBST (of both groups) in the 6th month showed a statistically significant difference (p˂0.05) (Fig. 4). The normal range for the saccharine test in the control group (14 normal participants) was established at 7.93 ± 1.59 min. Compared with the normal range, the saccharin test was higher in both groups than in the normal range over time (*P* < 0.05).

**Fig. 5:**
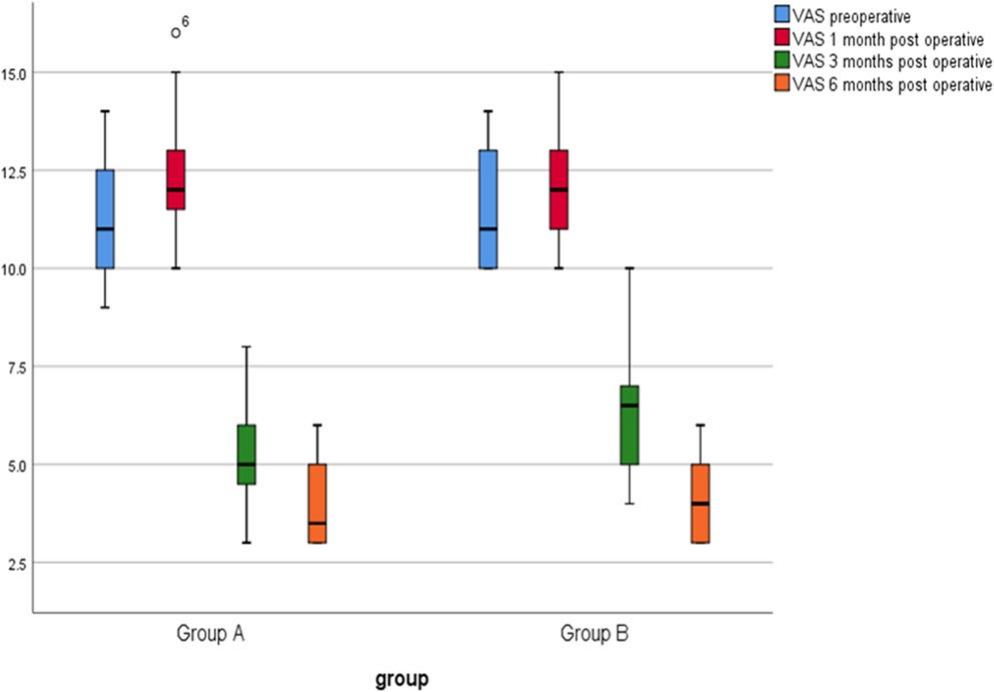
Boxplot for timeline measurements of VAS in both groups

Preoperatively, Group A had a median VAS-s of 10.06 (IQR = 10–12.75.75) and Group B had a median of 11.0 (IQR = 10–13), with no significant difference between the groups (*p* = 0.726). At one month postoperatively, both groups reported similar median scores (Group A: 12.0, IQR = 11.25–13.25; Group B: 12.1, IQR = 11–13), again without significant differences (*p* = 0.576). Improvements were observed at three months, where Group A’s median VAS score decreased to 5.0 (IQR = 4.25–6.25), while Group B’s median score was 6.0 (IQR = 5–7), approaching significance (*p* = 0.060). At six months, Group A reported a median of 3.5 (IQR = 3–5), while Group B’s median was 4.0 (IQR = 3–5), with no significant difference (*p* = 0.470). The differences within each group from preoperative to postoperative assessments showed significant improvements, particularly from preoperative to three months (*p* = 0.000 for both groups), indicating that both surgical interventions were effective in reducing symptoms over time (Fig. 5).

## Discussion

Patients with chronic rhinitis experience various annoying symptoms, including rhinorrhea, nasal obstruction, and nasal irritation, which have a detrimental impact on their quality of life. Various medications (and combinations of drugs) are prescribed and might report good responses; unfortunately, a considerable group of patients do not respond to these conservative measures (refractory rhinitis). These patients would be candidates for surgical treatments. Vidian neurectomy (VN) was described as a surgical option; the advancement in nasoendoscopic applications provided posterior nasal neurectomy (PN). PN is a selective subtype of VN that can effectively control the annoying symptoms of intractable rhinitis by limiting nasal mucosal hyperreactivity, improving nasal ventilation, and suppressing secretory activity. Also, PN could minimize the risks of irreversible complications of VN, including numbness of the palate and persistent irritating dry eyes [1–6, 9]

The posterior nasal nerve exits the sphenopalatine foramen (SPF) and is distributed (following the branches of the sphenopalatine vessels) to supply the inferior turbinate mucosa. Parasympathetic innervation increases secretomotor activities, while sensory innervation controls the sensitivity of the nasal mucosa. Previous research reported satisfactory results of PN; more than 50% of those studied patients were almost free of nasal symptoms [10, 11].

The ideal PN technique should effectively ablate the posterior nasal nerve, be conservative, report minimal/no complications (especially bleeding and crustation), give unique long-lasting good results among various centers, and be less costly. Although the available English literature shows different tools for endoscopic surgical PN (electrical and radiofrequency technologies) and reported accepted results, unfortunately, such a perfect maneuver is still, and debates exist [12–14]. Noticeably, the application of laser in PN was sparsely mentioned.

In the recent era of nasoendoscopy, Lasers have been proposed for various nasal procedures including PN. Laser has many advantages including limited tissue trauma and minimal bleeding. The physical parameters of the laser permit its applications in different ways. For example, the laser could shrink the inferior turbinate tissues with subsequent relief of nasal obstruction via its ability to induce sub-mucosal scarring and obliteration of the turbinate venous sinusoids. The diode laser (DL) has a special effect on the vascular tissues, is easily portable, and has a flexible fiber. Moreover, DL is considered the least expensive among all available lasers for rhinology applications. However, using DL needs experience and special precautions to avoid complications [7].

The current research was designed to compare the surgical outcomes of the diode laser posterior nasal neurectomy with or without posterior turbinectomy in patients with chronic refractory rhinitis. The study showed non-significant differences between the two groups during early follow up stage as regards postoperative complications (pain, crusting, epistaxis, and adhesions). Both groups showed high significant differences between the pre and postoperative results regarding rhinorrhea and nasal obstruction. However, comparing both groups showed no statistically significant difference regarding rhinorrhea, while nasal obstruction showed a significant difference (favorable towards group A).

VAS-s was employed for symptoms recording. After early postoperative elevation (which might be attributed to many factors: stresses, pains, discomfort, and crustations), the differences within each group (preop/postop assessments) showed significant improvement that became better over time, indicating that both surgical interventions were effective in control of symptoms.

MBST was applied in this work. Using the blue dye-stained saccharine, the test objectively studies the mucociliary clearance of the nasal mucosa. Comparing the preop/postop MB clearance time could provide valuable data regarding the mucociliary clearance and hence, the functional status of the nasal mucosa. The examiner could monitor the blue color at the pharynx and measure the transit time accurately without depending on the old subjective saccharine test [7]. According to the pharyngeal recovery of the saccharine (the mucociliary recovery), a delay in saccharine test time was observed a few weeks after surgery in the study group; this could be attributed to crustation and tissue edema [8]. By the 6th month, high significant differences were reported (in both groups) regarding the comparison with the preoperative time, but group B showed a better time than A. Noticeably, the comparison with the control group, MBST was higher in both groups over time.

The current study’s limitations include: (a) the relatively small sample size, (b) the relatively short follow-up period, (c) it represents a single-team/institution experience, and (d) the lack of objective assessment measures. Another important point is that patients could not be labeled as having vasomotor rhinitis, even if systemic allergy tests are negative; local allergic rhinitis cannot be excluded; this point is a usual obstacle regarding this subject. The authors recommend long-term, wide-scale studies for proper evaluation of the techniques, establishing its applicability, and testing durability and outcomes.

## Conclusion

Diode laser posterior nasal neurectomy and conservative posterior turbinectomy allowed the reduction of nasal hyperreactivity and improved the annoying symptoms of refractory vasomotor rhinitis. The procedure is less invasive, easily conducted, well-tolerated by patients, and has minimal complications. Although this study is a preliminary, short-term, single-institution study, the reported results suggest the feasibility of the procedure as a surgical option for refractory rhinitis. Further wide-scale research is encouraged.

## Data Availability

Data will be made available on reasonable request.
